# Phosphorylation, Dephosphorylation, and Multiprotein Assemblies Regulate Dynamic Behavior of Neuronal Cytoskeleton: A Mini-Review

**DOI:** 10.3389/fnmol.2018.00373

**Published:** 2018-10-08

**Authors:** Natalya Kurochkina, Manju Bhaskar, Sharda Prasad Yadav, Harish C. Pant

**Affiliations:** ^1^Department of Biophysics, The School of Theoretical Modeling, Washington, DC, United States; ^2^Neuronal Cytoskeletal Protein Regulation Section, National Institute of Neurological Disorders and Stroke, National Institutes of Health, Bethesda, MD, United States

**Keywords:** phosphorylation, ankyrin repeats, protein structure, neurodegeneration, Alzheimer’s disease

## Abstract

Cellular localization, assembly and abnormal aggregation of neurofilaments depend on phosphorylation. Pathological processes associated with neurodegeneration exhibit aberrant accumulation of microtubule associated aggregated forms of hyperphosphorylated neuronal protein tau in cell bodies. These processes are critical for the disease progression in patients suffering from Alzheimer’s disease, Parkinson’s disease, and Amyotrophic Lateral Sclerosis. In healthy cells, tau is localized in axons. Topographic regulation suggests that whereas the sites of synthesis of kinases and neurofilaments are the cell bodies, and sites of their functional assemblies are axons, phosphorylation/dephosphorylation are the key processes that arrange the molecules at their precise locations. Phosphorylation sites in the dynamic developmental and degenerative processes differ. Not all these processes are well understood. New advancements identify epigenetic factors involved in AD which account for the influence of age-related environment/genome interactions leading to the disease. Progress in proteomics highlights previously found major proteins and adds more to the list of those involved in AD. New key elements of specificity provide determinants of molecular recognition important for the assembly of macromolecular complexes. In this review, we discuss aberrant spatial distribution of neuronal polypeptides observed in neuropathies: aggregation, association with proteins of the neuronal cytoskeleton, and phosphorylation dependent dynamics. Particularly, we emphasize recent advancements in understanding the function and determinants of specific association of molecules involved in Alzheimer’s disease with respect to the topographic regulation of phosphorylation in neuronal cytoskeleton and implications for the design of new therapies. Further, we address the role of various filament systems in maintenance of the shape, rigidity and dynamics of the cytoskeleton.

## Introduction

**FIGURE 1:**
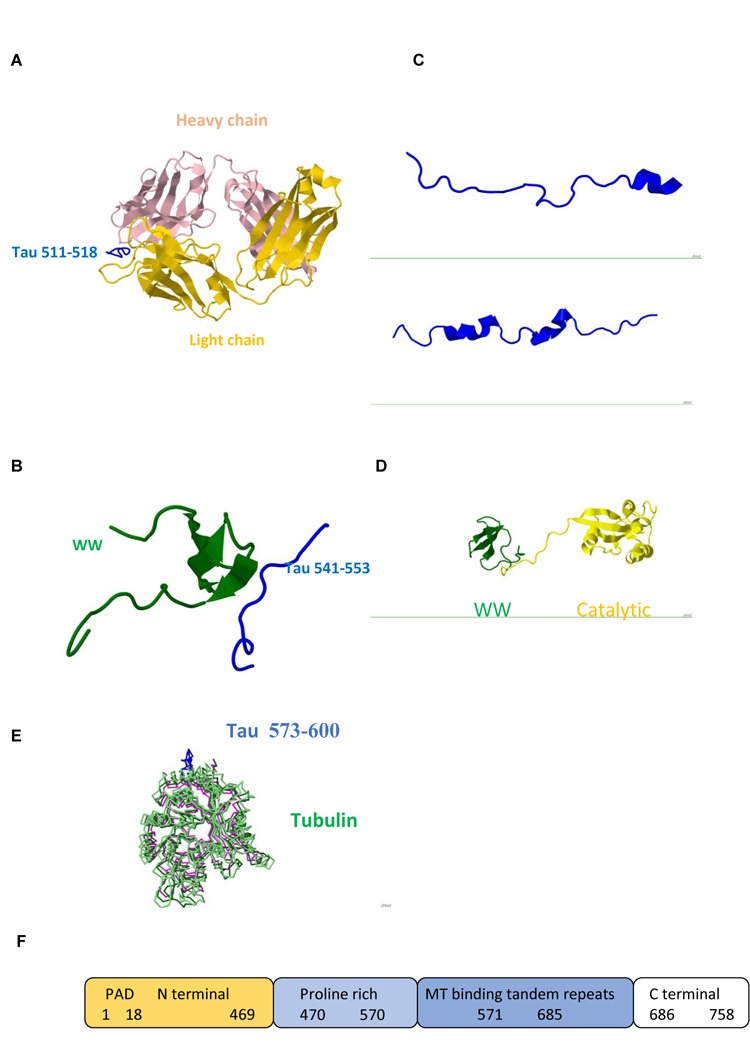
Tau protein **(A)** Triply phosphorylated at S202/T205/S208 Tau peptide 202–209/511–518 (blue) in complex with anti-tau antibody AT8 (Light chain gold and Heavy chain pink) /Pdb entry 5e2w/. **(B)** Fragment 541–553 bound to the WW domain (green) of PIN1 / Pdb entry 1i8h/. **(C)** Fragments 292–319/608–635 /5n5b/ and 254–290/571–607 /Pdb entry 5n5a/ bound to F-actin. **(D)** PIN1 WW (green) and catalytic (yellow) domains / Pdb entry 1nmv/. **(E)** Fragment 573–600 (blue) /Pdb entry 6cvn/ bound to tubulin (green, magenta). **(F)** Domain structure; PAD, Phosphatase Activating Domain.

Cellular localization, assembly and abnormal aggregation of NFs depend on phosphorylation (Boumil et al., 2018). Pathological processes associated with neurodegeneration exhibit aberrant accumulation of MT associated aggregated forms of hyperphosphorylated neuronal protein tau in the cell bodies (Binukumar et al., 2013). These processes are critical for the disease progression in patients suffering from the AD, PD, and Amyotrophic Lateral Sclerosis (ALS). In the healthy cells, NFs and tau are localized in the axons. Proteolysis of the cell surface receptor and cell adhesion molecule APP results in the production of amyloid peptides (Roy and Rauk, 2005). Aβ peptide induces alterations in axon to soma (retrograde) and soma to axon (anterograde) traffic of tau isoforms that regulates distribution of tau in neurons, F-actin remodeling, reduced MT dynamics, and impairment of astrocytic glutamate transport (Kanaan et al., 2013; Zempel et al., 2017). Distribution of NFs and tau is regulated by posttranslational modifications (Shukla et al., 2012; Kim et al., 2014) and interactions with cellular components such as AIS proteins ankyrin G (AnkG), EB1 and GSK-3β (Zempel et al., 2017) or PP2A and peptidyl-prolyl *cis*/*trans* isomerase PIN1 (**Figure 1**; Rudrabhatla et al., 2009). Tau and APP intracellular fragments alter gene transcription (Multhaup et al., 2015). Phosphorylation and dephosphorylation by kinases/phosphatases are key regulatory processes that we target to provide effective preventive and treatment options. AD brains are subject to neuroinflammation regulated by microglia and immune cells in the central nervous system (Mastroeni et al., 2017). Microglia carry out both beneficial and harmful functions: plaque removal or release of neurotoxic inflammatory materials. Although genetic associations exist which point to variations in genes of glia residing proteins, further studies identify epigenetic factors involved in AD which account for influence of age-related environment/genome interactions leading to the disease. Progress in proteomics adds new proteins involved in AD (Gozal et al., 2009; Guzmán-Martinez et al., 2013; Robinson et al., 2017). Cytoskeletal proteins interact with each other and assemble in filaments in a highly specific manner; new key elements of specificity provide determinants of molecular recognition in the assembly of macromolecular complexes (Kurochkina and Iadarola, 2015). Understanding the function of kinases, phosphatases, and their targets outlines important implications and insights how to prevent and cure progressive neurological diseases.

In this review, we discuss aberrant spatial distribution of neuronal polypeptides observed in neuropathies: aggregation, association with proteins of the neuronal cytoskeleton, and phosphorylation dependent dynamics. Particularly, we emphasize recent advancements in understanding the function and determinants of specific association of the molecules involved in AD with respect to the topographic regulation of phosphorylation in neuronal cytoskeleton and implications for the design of new therapies. Further, we address the role of various filament systems in maintenance of the shape, rigidity and dynamics of the cytoskeleton.

## Phosphorylation and Dephosphorylation as Key Factors of Protein Dynamics, Association, Neuronal Development, and Pathological Processes

### Cytoskeletal Proteins

Filament systems like MTs, microfilaments, and intermediate filaments (IFs) comprise cell cytoskeleton as key elements regulating cell shape, rigidity and dynamics (Gruenbaum and Aebi, 2014). Structure of fibrous and globular filamentous proteins, assembly of filaments, and movement of motors are important for cell dynamics and function (Siddiqui and Straube, 2017). Cytoskeleton not only provides cell support but is also an important regulator of cell highly regular organization, mobility, reaction pathways, and response to extreme conditions (Unsain et al., 2018). Interactions of cytoskeletal components with each other and with surrounding proteins are highly specific, tightly regulated and drive functional assemblies of the multiprotein complexes (Kurochkina and Iadarola, 2015).

**FIGURE 2:**
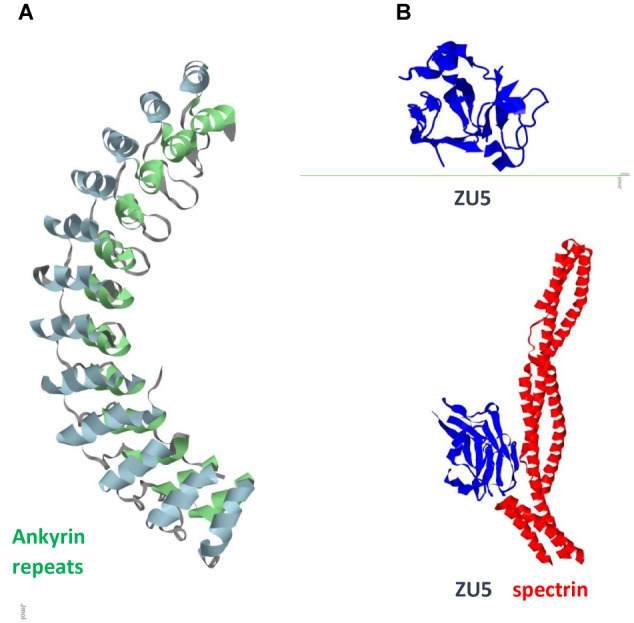
Ank1 **(A)** Ankyrin repeats domain / Pdb entry 1n11/. Inner row helices green; outer row helices blue. **(B)** Spectrin binding ZU5 domain/C-terminal fragment ZU5-ANK (blue) / Pdb entry 3f59/ and its complex with spectrin repeats (red) / Pdb entry 3kbt/.

Studies of neuron specific cytoskeletal proteins such as tau and light (NFL), medium (NFM), and heavy (NFH) subunits of NFs show that phosphorylation plays important role in their function and dynamics. Phosphorylation stabilizes NFs, protects from proteolysis, and promotes calcium mediated assembly of NF subunits and cytoskeletal components in axons, whereas dephosphorylation results in more dynamic chains which repair and regenerate less mobile chains (Rudrabhatla et al., 2009; Boumil et al., 2018). Tau regulates MT polymerization and contains multiple binding sites. Its dynamic behavior is reflected in its structure. Large portions of the molecule are disordered (**Figure 1**) and are candidates for binding other ligands. Carrying PxxP sequence, tau mono-, di- and tri-phosphorylated peptides exhibit polyproline II conformation in complex with antibody (**Figure 1A**) and PIN1 (**Figure 1B**). Interactions of two Pin1 domains, WW substrate recognition and catalytic (**Figure 1D**), with each other, PP2A and other proteins contribute to its function (Wang et al., 2017). Tau obtains helical conformation upon binding to tubulin and F-actin (Kellogg et al., 2018; **Figure 1C**). All six human tau isoforms show common mechanism of tau/PAD (**Figure 1F**) conformational mobility associated with axonal transport inhibition (Cox et al., 2016). Both NFs and tau associate with MTs and produce huge complexes (**Figure 1E**) which are tethered to a membrane. Ankyrin repeats target proteins to the plasma membrane and link membrane proteins to the actin/spectrin cytoskeleton (**Figure 2**).

### Symmetry/Polarization in Neuronal Development

Neuron-specific cytoskeleton gives neuronal cell its shape. Unique features of the neuron with cell body in the central and meters long axons in the peripheral nervous system require specific structural components and maintenance (Binukumar et al., 2013). The asymmetric MT cytoskeleton plays a key role in axon-dendrite specification during the development and polarized protein sorting in mature neurons. The distribution of neuronal proteins to axons and dendrites depends on joint action of MT-binding proteins CRMP, directed motors (kinesin UNC-104/Kif1A) (Kanaan et al., 2013), and diffusion barriers (ankyrin) (Maniar et al., 2012).

### Phosphorylation/Dephosphorylation in Pathologies

Pathological hyperphosphorylated and aggregated tau is surrounded by more than 150 proteins as shown by studies of neurofibrillary tangles (Wang et al., 2005). Many efforts focus on abnormal aggregation of Aβ, NFs and tau. However, the impairment of the function of tau, NFs, and other proteins in topographic regulation of phosphorylation could provide keys to pathology (Lane et al., 2012). Tau and NF colocalize and co-immunoprecipitate with PSD95 regulating the interaction of PSD95 with NMDAR subunits. Axonal protein tau has a dendritic function: postsynaptic targeting of the Src kinase Fyn. Fyn phosphorylates the NR subunit 2 of NMDA receptors (NMDARs) and promotes its interaction with the PSD95 (Ittner et al., 2010). NMDARs, for instance, mediate excitatory glutamatergic signals from ON and OFF bipolar cells in distinct subliminae of the inner plexiform layer to retinal ganglion cells. Tetrameric structure of NMDAR embraces two NR1 and two NR2 subunits. Localization of NR2A/NR1C2 splice variant and PSD93 /PSD95 of MAGUK to the PSD OFF synapses whereas NR2B/NR1C2 splice variant and SAP102/MAGUK to the ON synapses perisynaptically suggests specific roles for various receptor isoforms (Zhang et al., 2016). NMDAR is regulated by Cdk5 phosphorylation (Li et al., 2001). MT-binding protein CRMP (UNC-33) being similar to tau is also involved in dynamic regulation of MTs, and its isoforms expressed during brain oncogenesis carry out signaling function in axon outgrowth and guidance (Bretin et al., 2005; Meng et al., 2016). Cdk5, GSK3β, and Pin1 regulate CRMP2A, major isoform of CRMP (Maniar et al., 2012). Unlike tau, CRMP2, regulated by posttranslational modifications, actively engages in protein endocytosis and vesicular cycling. Aβ-induced modifications of CRMP2 could possibly result in impaired axonal transport and synapse loss: pathological processes associated with AD (Hensley and Kursula, 2016; Yang et al., 2017). CRMP2 is targeted as possible agent that could prevent or delay the progression of AD.

Hyperphosphorylation with involvement of Cdk5, GSK3β and other kinases and oxidation contribute to AD pathogenesis (Khanna et al., 2012). Phosphorylation of neuronal cytoskeletal proteins is restricted selectively in axonal but not in cell body compartment under physiological conditions. However, under pathological state, it is deregulated, and occurs aberrantly in the cell body compartment as well. Phosphorylation sites in the dynamic developmental and degenerative processes differ (Yu et al., 2009). Strategies to treat pathologies target posttranslational modifications and mediation of localization, dynamics, and assembly.

## Domains of Specific Multiprotein Assemblies: Ankyrin Repeats

Ankyrin repeats are important scaffold components that mediate shape, rigidity, dynamics of association, and function of the multiprotein assemblies. In mammals, ankyrins are encoded by three different genes, ankyrin R (ANK1), ankyrin B (ANK2/ANKB), and ankyrin G (ANK3/ANKG), all of which have similar structure and function, and undergo alternative splicing to generate multiple isoforms (Kordeli et al., 1995). Epigenetic wide association studies identified ANK1 (**Figure 2**) as one of the key risk factors for AD (Chi et al., 2016; Mastroeni et al., 2017). ANK1 is also implicated in type 2 diabetes (Imamura et al., 2012). Microglia is the source of ANK1 and no elevated expression of ANK1 occurs in CA1 astrocytes or neurons. Changes in expression of 13 out of 14 ankyrin repeats proteins in AD neurons with downregulated AnkRD18 and upregulated AnkRD34, increased ANK1 in PD microglia, elevated AnkRD36/ANKRD52/ANKRD18CP in AD astrocytes, and overexpressed Ankle2 in PD microglia suggest that this involvement is not disease specific but a process related to neuroinflammation. Association of ANK1 gene methylation changes with AD (Lunnon et al., 2014) shows how epigenetic mechanisms could provide a link to age-related inflammation and highlights this gene as an AD risk factor.

Progress in proteomics reveals AnkB and AnkG involvement in AD (Gozal et al., 2009). Giant long ANKB isoform in Drosophila shows involvement in synapse stability and links to neurodegeneration (Pielage et al., 2008). Knockout of the long AnkG isoform results in neuroanatomical defects in mice (van der Werf et al., 2017) and impaired filtering of cellular components (Sun et al., 2017). AnkG is the site of clustering of Na^+^ and K^+^ channels and their interactions with numerous modulators (Gennarini et al., 2017). AnkG, site of assembly of Na^+^ /K^+^-ATPase, together with other cytoskeletal proteins (GABA A receptor gephyrin) makes connections to membrane proteins associated with lipid rafts (Dalskov et al., 2005). AnkG may be considered the most important AIS constituent. The AIS is a specialized compartment in neurons that resides between axonal and somatodendritic domains where the assembly of neuronal IFs takes place. It serves as a site of action potential firing and helps maintain neuron polarity. It also acts as a submembrane diffusion barrier that restrict the mobility of plasma membrane components (Bennett, 1992; Winckler et al., 1999) and an intracellular selective filter for the transport of organelles and molecules between these domains through the cytoplasm. AnkG integrates proteins that function in three layers of the AIS: the plasma membrane (outermost surface), submembrane cytoskeleton (middle layer), and inner AIS shaft (cytoplasmic region), and acts as its main organizer. Advancements in deciphering structure of ankyrins, phosphorylation states and role in assembly of the multiprotein complexes help to understand the mechanisms and suggest new studies (Zhang et al., 1998; Chen et al., 2017).

## Conclusion

Progress in understanding the role of phosphorylation/dephosphorylation in regulation of spatial distribution and transport of proteins involved in neurodegenerative diseases brings new approaches and evolvement of new treatments. Understanding MT dynamics, posttranslational modifications, specific distribution and assembly of cellular components, and signaling which links neuronal dysfunction to neuropathies leads to the development of new therapies (Yeh et al., 2004; Shukla et al., 2012; Kanaan et al., 2013). Aβ accumulation can be treated with amyloid removal strategies such as one utilizing DARPins (Hanenberg et al., 2014). Ankyrin G vaccination is tested for the reduction of Aβ and lowering toxicity (Santuccione et al., 2013). Ankyrin repeats are interaction sites of phosphorylation dependent dynamic assembly of proteins and nucleic acids (Hall et al., 2018) including those involved in transcription regulation (Kohda et al., 2016) and signaling, and present promising targets for the design of new drugs.

## Author Contributions

All authors listed have made a substantial, direct and intellectual contribution to the work, and approved it for publication.

## Conflict of Interest Statement

The authors declare that the research was conducted in the absence of any commercial or financial relationships that could be construed as a potential conflict of interest.

## Abbreviations

Aβ: Amyloid-β
AD: Alzheimer’s disease
AIS: axon initial segment
APP: amyloid precursor protein
CRMP: Collapsin response mediator protein
GSK-3β: glycogen synthase kinase
DARPin: designed ankyrin repeats protein
MT: microtubule
NF: neurofilament
NMDAR: *N*-methyl-di-aspartate receptor
PD: Parkinson’s disease
PIN1: peptidylprolyl *cis*/*trans* isomerase NIMA-interacting 1
PP2A: protein phosphatase 2A
PSD95: postsynaptic density protein 95.
